# Supplementary material for: Fibrinolytic Dysregulation in Regional Hemostasis During Liver Transplantation: A Viscoelastometry-Based Pilot Study
Source: J Clin Med. 2025 Apr 24;14(9):2925. doi: 10.3390/jcm14092925 (PMC12072297; doi:10.3390/jcm14092925)
Supplement: Supplementary file 1 [file jcm-14-02925-s001.zip › jcm-3586173-supplementary.pdf]

Test	$CaCl_2$	Activator	Modificator	Polybrene*	Clinical use
EX-test	✓	Tissue factor	-	✓	Assessment of extrinsic pathway
FIB-test	✓	Tissue factor	Cytochalasin-D+ Glycoprotein IIb/IIIa inhibitor	✓	Assessment of functional fibrinogen
TPA-test	✓	Tissue factor	tissue plasminogen activator	✓	Detection of antifibrinolytics
IN-test	✓	Ellagic acid	-	✗	Assessment of Intrinsic pathway
RVV-test	✓	Russel vipera venom	-	✗	Detection of Anti-Xa inhibitor effect
ECA-test	✗	Ecarin (Arab efa venom)	-	✓	Detection of thrombin inhibitor effect

Table S1. The composition and clinical application of the ClotPro tests we used

*Polybrene (hexadimethrine bromide) neutralizes any heparin that may be present in the sample

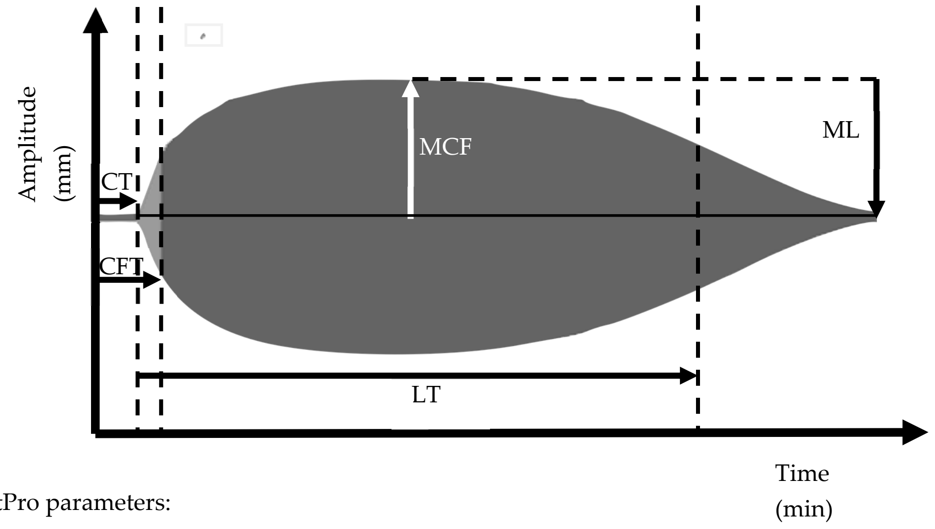

Figure S1. ClotPro parameters:

Clotting Time (CT): the time elapsed from the start of the measurement until a 2 mm clot forms.

Clot Formation Time (CFT): the time elapsed until an amplitude of 20 mm is reached.

Maximum Clot Firmness (MCF): the greatest deviation measured.

Maximum Lysis (ML): the greatest reduction in amplitude measured within one hour after reaching the MCF.

In the TPA test, the Lysis Time (LT): the time elapsed from reaching CT until the amplitude decreases to 50% of the MCF.

S1-systemic sample			Identical group		Hyperfibrinolytic group		Fibrinolytic shutdown group		p
			mean	SD	mean	SD	mean	SD	
ClotPro	EX-test	CT	68.63	12.526	68.13	19.394	64.5	8.976	0.828
		CFT	77.89	40.924	124.25	158.953	54.25	15.872	0.534
		MCF	54.89	9.86	54.75	14.31	61	5.398	0.383
		ML	6.79	3.489	7.25	4.132	5.88	3.314	0.724
	IN-test	CT	166.16	23.871	177.13	52.9	169.25	13.371	0.957
		CFT	107.63	57.329	150.63	176.945	68.88	23.739	0.330
		MCF	52.05	9.997	53.5	14.061	57.38	6.046	0.435
		ML	5.47	2.951	5.13	2.85	5.25	3.37	0.971
	FIB-test	CT	75.95	19.648	131	190.095	68	18.739	0.557
		MCF	18.26	6.806	19.63	9.826	22.63	6.632	0.275
		ML	0.11	0.315	0.14	0.378	0	0	0.586
	TPA-test	CT	56.21	11.612	55.75	18.422	53.38	9.226	0.762
		MCF	28.26	12.48	31.13	18.027	36.75	9.208	0.272
		LT	195.79	63.897	658.13	1190.912	239	72.832	0.099
		ML	93.42	3.043	95.5	2.673	95.5	1.414	0.130
	RVV-test	CT	80.95	21.128	72.25	22.714	81.13	20.153	0.441
		CFT	91.32	56.309	129.38	160.536	59.88	28.618	0.589
		MCF	53.89	9.910	54.25	14.17	59.38	6.391	0.472
		ML	5.16	3.5	5.13	3.091	4.75	2.435	0.846
	ECA-test	CT	79.63	13.263	87.5	21.065	82.12	14.721	0.547
		CFT	100.74	28.4	134.88	115.011	94.13	13.282	0.892
		MCF	58.68	9.673	57.13	14.691	63.38	5.236	0.496
		ML	0.05	0.229	2.13	6.01	0	0	0.544
BGA		pH	7.41	0.046	7.44	0.081	7.43	0.0307	0.508
		Lactate	0.85	0.301	0.83	0.437	0.88	0.320	0.799
		BE	0.50	2.469	2.76	5.312	1.14	1.648	0.556
		Bicarbonate	25.33	1.827	26.96	3.901	25.46	0.811	0.664
		Ca^{2+}	0.91	0.144	0.976	0.190	0.97	0.136	0.784
CCT		PLT	140.47	137.455	171.63	166.983	134	60.429	0.659

PT (s)	19.63	5.281	24.26	13.410	23.63	14.226	0.783
INR	1.62	0.448	2.44	2.286	2.10	1.599	0.741
aPTT	52.68	18.118	64.39	25.756	57.34	23.281	0.461
Fibrinogen	2.52	0.943	2.73	1.406	3.44	0.804	0.113
D-dimer	1.31	1.862	1.29	1.941	1.67	1.958	0.718
FV	44.60	29.572	37.05	25.011	40.8	15.9956	0.775
FVII	56.72	30.971	58.96	29.995	51.4	13.0305	0.834
FX	62.8	27.995	53.6	16.0663	56.713	17.2918	0.830
FXIII	121.51	33.4792	122.588	47.2197	114.937	17.8034	0.920

Table S2. Parameters of the S1 sample („before surgery”) in the three subgroups.

aPTT: activated partial thromboplastin time, BE: base excess, BGA: blood gas analysis, Ca^{2+}: ionized calcium, CCT: conventional coagulation test, CFT: clot formation time, CT: clotting time, FV: factor V, FVII: factor VII, FX: factor X, FXII: factor XIII, INR: international normalized ratio, LT: lysis time, MCF: maximum clot firmness, ML: maximum lysis, PLT: platelet, PT: prothrombin time, SD: standard deviation.

S2-systemic sample			Identical group		Hyperfibrinolytic group		Fibrinolytic shutdown group		p
			mean	SD	mean	SD	mean	SD	
ClotPro	EX-test	CT	66.84	14.561	64	13.005	65.25	7.833	0.869
		CFT	72.42	38.028	84.13	59.468	51.5	16.018	0.370
		MCF	56.95	8.91	55.75	12.033	61.63	5.449	0.407
		ML	19.21	28.736	36	43.736	5.25	5.392	0.253
	IN-test	CT	162.63	33.322	227.38	158.575	145.63	22.36	0.324
		CFT	100.74	51.122	120.57	110.351	66.13	19.759	0.271
		MCF	53.95	8.638	46.63	22.513	59.88	5.384	0.234
		ML	15.16	24.654	34.29	43.235	3.63	3.42	0.103
	FIB-test	CT	72.47	17.973	65.63	18.769	69.75	17.417	0.618
		MCF	18.37	7.403	17.5	8.992	20.38	6.718	0.586
		ML	25.68	34.192	25	40.086	5.88	7.791	0.496
	TPA-test	CT	54.58	14.634	49.25	14.577	54.5	5.976	0.527
		MCF	28.68	12.858	28.63	12.27	35.88	7.453	0.159
		LT	192.89	73.022	205	55.755	227.38	36.328	0.128
		ML	93.68	2.849	93.38	4.207	95.63	1.302	0.222
	RVV-test	CT	74.42	16.584	137.88	187.287	68.38	26.817	0.663
		CFT	90.74	50.081	115.71	124.346	60	22.772	0.330
		MCF	55.16	8.533	48.38	22.456	60.25	5.874	0.370
		ML	11.63	22.157	30.14	43.483	3.62	3.889	0.245
	ECA-test	CT	84.89	18.15	86.13	16.366	84	13.027	0.913
		CFT	92.89	25.588	115.12	66.297	89.88	8.643	0.644
		MCF	56.89	9.763	53.5	14.687	62.38	5.902	0.277
		ML	29.42	38.356	39.25	47.743	7	11.148	0.303
BGA		pH	7.38	0.044	7.42	0.062	7.70	0.028	0.091
		Lactate	1.44	0.417	1.58	0.618	1.68	0.621	0.640
		BE	-1.84	2.424	0.49	4.181	-2.04	1.908	0.090
		Bicarbonate	23.69	1.772	25.41	3.078	23.61	1.322	0.095
		Ca^{2+}	1.05	0.184	0.86	0.208	0.88	0.121	0.340
CCT		PLT	166.17	147.249	176.38	156.407	134.13	57.878	0.989

PT (s)	20.64	4.737	24.53	9.271	22.55	8.368	0.676
INR	1.70	0.401	2.04	0.781	1.86	0.711	0.664
aPTT	50.54	17.733	68	28.233	56.86	21.964	0.195
Fibrinogen	1.99	0.863	1.91	1.137	2.79	1.090	0.091
D-dimer	2.74	1.733	2.199	1.825	3.65	2.662	0.432
FV	37.56	24.296	27.81	18.665	33.55	20.074	0.617
FVII	49.50	23.783	50.21	23.253	49.09	15.296	0.890
FX	54.1	22.523	45.01	15.477	55.4	13.333	0.324
FXIII	111.55	37.194	109.59	49.647	114.24	38.139	0.767

Table S3. Parameters of the S2 sample („hepatectomy”) in the three subgroups.

aPTT: activated partial thromboplastin time, BE: base excess, BGA: blood gas analysis, Ca^{2+}: ionized calcium, CCT: conventional coagulation test, CFT: clot formation time, CT: clotting time, FV: factor V, FVII: factor VII, FX: factor X, FXII: factor XIII, INR: international normalized ratio, LT: lysis time, MCF: maximum clot firmness, ML: maximum lysis, PLT: platelet, PT: prothrombin time, SD: standard deviation.

S3-systemic sample			Identical group		Hyperfibrinolytic group		Fibrinolytic shutdown group		p
			mean	SD	mean	SD	mean	SD	
ClotPro	EX-test	CT	67.42	14.057	63.38	18.624	66	7.709	0.564
		CFT	81.11	53.854	108.63	86.723	60.25	17.621	0.437
		MCF	54.84	8.434	51.63	15.023	58.75	6.585	0.363
		ML	20.63	28.214	36.13	45.177	18.88	30.418	0.778
	IN-test	CT	169.11	37.728	171.38	58.931	189.75	72.51	0.685
		CFT	120.78	87.411	190.75	192.892	86.38	34.017	0.158
		MCF	50.63	11.744	47.88	16.78	56	7.616	0.417
		ML	11.32	16.327	32.13	41.927	15.25	24.341	0.817
	FIB-test	CT	80.68	21.124	68.88	17.365	80.75	20.331	0.355
		MCF	14.68	6.174	15.5	9.243	16.88	7.298	0.848
		ML	24.84	34.835	11.75	30.881	31	41.366	0.303
	TPA-test	CT	55.84	14.713	60.63	27.26	51.25	11.411	0.707
		MCF	26.11	12.192	24.88	18.326	32.75	12.395	0.358
		LT	189.37	60.757	199	90.51	217.13	77.578	0.699
		ML	92.79	3.172	92.75	11.132	94.88	2.031	0.111
	RVV-test	CT	79.32	17.945	77.88	16.703	121.75	136.959	0.981
		CFT	105.42	74.966	136	141.98	77.88	39.628	0.574
		MCF	54.05	8.554	50.75	13.792	57.38	8.297	0.331
		ML	9.58	18.839	27	42.541	11.25	18.911	0.920
	ECA-test	CT	84.42	18.112	85.13	20.23	94.25	11.817	0.195
		CFT	104	44.208	138.88	132.042	91	4.899	0.767
		MCF	55.32	9.798	51.38	17.936	58.5	7.091	0.674
		ML	35.68	40.669	36.75	48.561	31.5	44.753	0.863
BGA		pH	7.31	0.054	7.36	0.058	7.32	0.0378	0.303
		Lactate	2.51	1.258	2.63	0.981	2.76	0.826	0.518
		BE	-3.81	2.612	-1.81	3.564	-3.95	1.606	0.425
		Bicarbonate	21.77	2.118	23.08	2.621	21.66	1.112	0.568
		Ca^{2+}	0.92	0.270	0.93	0.086	1.00	0.183	0.778
CCT		PLT	142.74	134.379	159.75	165.673	154	51.069	0.269

PT (s)	24.04	8.864	27.99	17.025	28.59	17.089	0.910
INR	1.99	0.751	2.5	1.896	2.78	2.567	0.907
aPTT	59.22	26.909	70.39	52.909	67.54	39.093	0.799
Fibrinogen	1.69	0.794	2.05	1.597	2.16	0.867	0.412
D-dimer	4.85	1.792	3.41	1.884	4.63	1.983	0.388
FV	31.10	24.067	29.11	21.296	30.88	14.347	0.860
FVII	43.1	22.510	46.93	22.758	39.29	14.774	0.713
FX	46.75	23.195	42.4	14.779	44.95	13.920	0.943
FXIII	103.40	36.799	111	56.254	95.25	30.190	0.827

Table S4. Parameters of the S3 sample („anhepatic phase”) in the three subgroups.

aPTT: activated partial thromboplastin time, BE: base excess, BGA: blood gas analysis, Ca^{2+}: ionized calcium, CCT: conventional coagulation test, CFT: clot formation time, CT: clotting time, FV: factor V, FVII: factor VII, FX: factor X, FXII: factor XIII, INR: international normalized ratio, LT: lysis time, MCF: maximum clot firmness, ML: maximum lysis, PLT: platelet, PT: prothrombin time, SD: standard deviation.

S4-systemic sample			Identical group		Hyperfibrinolytic group		Fibrinolytic shutdown group		p
			mean	SD	mean	SD	mean	SD	
ClotPro	EX-test	CT	70.76	15.69	69.14	16.737	62	13.48	0.374
		CFT	77.71	42.497	84.25	51.079	58.87	24.369	0.463
		MCF	55.06	7.949	54.5	10.461	59.25	7.265	0.437
		ML	4.06	3.288	15.5	29.592	3.38	2.387	0.523
	IN-test	CT	208	50.208	222.25	77.667	191.5	38.326	0.729
		CFT	129.11	82.719	157.88	129.801	92.38	43.563	0.38
		MCF	52.11	9.145	50.25	12.578	55.13	8.357	0.703
		ML	3.11	2.654	9.25	18.172	2.87	2.416	0.836
	FIB-test	CT	78	24.324	70.38	18.57	71.38	18.322	0.675
		MCF	14.5	5.25	18.12	8.167	17.25	6.923	0.471
		ML	0.5	1.098	11.63	32.08	2.13	2.642	0.245
	TPA-test	CT	59.06	13.414	54	11.747	53.38	14.392	0.683
		MCF	26.72	10.56	30.57	13.575	33.75	11.498	0.324
		LT	192.5	56.082	206.29	58.708	220.5	56.419	0.531
		ML	93.28	3.196	93.57	4.198	95	2.07	0.321
	RVV-test	CT	97.28	22.557	99.5	24.437	82.13	25.227	0.273
		CFT	103.61	66.087	118.62	97.202	73.13	34.257	0.463
		MCF	53.72	8.428	53.88	10.895	57.63	7.981	0.523
		ML	3.39	2.725	6.25	7.285	2.75	2.053	0.569
	ECA-test	CT	94.78	37.671	85.5	21.173	84.87	13.432	0.845
		CFT	100.56	35.125	134	120.145	93.75	16.508	0.972
		MCF	57.44	8.494	55.25	13.625	60.38	7.577	0.674
		ML	4.28	16.197	24	44.443	12.25	29.207	0.781
BGA		pH	7.35	0.063	7.37	0.057	7.35	0.044	0.640
		Lactate	2.89	1.555	2.79	1.958	3.23	0.756	0.268
		BE	-4.44	4.326	-3.39	3.599	-4.74	1.450	0.651
		Bicarbonate	21.95	3.263	22.6	2.666	21.59	1.072	0.652
		Ca^{2+}	1.00	0.199	0.93	0.111	1.08	0.214	0.183
CCT		PLT	154.89	129.357	134.25	117.474	147.38	72.091	0.680

PT (s)	30.82	12.119	36.85	21.967	34.35	16.677	0.890
INR	2.73	1.542	3.08	1.868	3.35	2.281	0.890
aPTT	80.48	42.605	82.34	38.403	83.11	43.771	0.854
Fibrinogen	1.5	0.684	1.74	0.980	1.91	1.092	0.639
D-dimer	5.30	1.671	4.12	2.152	6.571	1.442	0.043
FV	18.3	19.107	17.46	16.183	19.18	13.602	0.885
FVII	37.47	19.277	39.7	19.087	34.61	10.612	0.875
FX	36.68	17.727	31.88	15.359	36.95	11.234	0.628
FXIII	95.4	35.642	102.65	51.283	89.8	38.425	0.880

Table S5. Parameters of the S4 sample („neohepatic phase”) in the three subgroups.

aPTT: activated partial thromboplastin time, BE: base excess, BGA: blood gas analysis, Ca^{2+}: ionized calcium, CCT: conventional coagulation test, CFT: clot formation time, CT: clotting time, FV: factor V, FVII: factor VII, FX: factor X, FXII: factor XIII, INR: international normalized ratio, LT: lysis time, MCF: maximum clot firmness, ML: maximum lysis, PLT: platelet, PT: prothrombin time, SD: standard deviation.

S5-systemic sample			Identical group		Hyperfibrinolytic group		Fibrinolytic shutdown group		p
			mean	SD	mean	SD	mean	SD	
ClotPro	EX-test	CT	68.47	11.988	69.25	14.119	68.25	12.992	0.961
		CFT	79.63	36.997	83.25	54.691	57.88	22.944	0.435
		MCF	55.11	7.549	56	10.365	59.88	6.833	0.377
		ML	4.79	6.803	2.25	3.955	3	2.563	0.266
	IN-test	CT	206.28	49.744	267.25	146.702	246	165.218	0.603
		CFT	120.78	57.202	124.86	96.505	102.88	69.138	0.545
		MCF	51.89	7.97	46.5	20.84	56	8.635	0.507
		ML	3.61	3.22	2.29	2.498	2.88	2.232	0.634
	FIB-test	CT	79.63	18.419	68.57	17.252	82.25	22.575	0.412
		MCF	14.05	4.116	17.14	7.647	16.63	5.975	0.556
		ML	4.47	19.5	0	0	0.13	0.354	0.613
	TPA-test	CT	55.47	10.824	54.38	11.686	60.63	11.999	0.698
		MCF	26.42	10.068	39.25	12.77	37.63	13.092	0.230
		LT	187.11	62.172	1115.88	1536.493	269.25	149.046	0.060
		ML	93.53	2.091	71.75	44.009	84	31.951	0.495
	RVV-test	CT	100.89	25.443	165.62	176.178	178.88	267.609	0.328
		CFT	102.89	50.669	167.71	231.847	87.5	62.221	0.576
		MCF	53.74	7.971	46.38	22.181	57.13	9.219	0.520
		ML	3	2.728	1.63	2.326	2.63	2.066	0.434
	ECA-test	CT	94.47	20.81	84.75	9.239	105	28.899	0.257
		CFT	103.84	40.236	120.88	73.283	106.37	40.673	0.993
		MCF	56.53	8.656	58.5	10.941	62.5	5.477	0.177
		ML	5.11	22.253	0.13	0.354	0	0	0.586
BGA		pH	7.35	0.043	7.38	0.039	7.32	0.056	0.083
		Lactate	2.96	2.361	2.4	1.577	3.76	2.151	0.289
		BE	-4.94	2.454	-2.83	3.615	-7.09	4.181	0.104
		Bicarbonate	21.92	2.300	22.74	1.813	20.35	2.501	0.152
		Ca^{2+}	0.93	0.178	0.96	0.151	0.91	0.293	0.968
CCT		PLT	157.72	131.398	160.5	117.313	194.87	80.804	0.307

PT (s)	35.38	16.020	39.38	18.835	34.4	14.015	0.951
INR	2.95	1.359	3.29	1.595	2.96	1.436	0.963
aPTT	86.84	43.693	97.9	41.816	90.64	47.073	0.708
Fibrinogen	1.46	0.520	1.85	0.593	1.91	0.631	0.098
D-dimer	5.05	1.452	5.96	1.355	5.57	2.185	0.401
FV	14.09	16.888	10.25	7.625	14.75	13.553	0.850
FVII	35.58	18.357	34.25	12.950	32.4	10.424	0.864
FX	33.19	18.783	29.38	14.03	32.58	13.419	0.797
FXIII	83.98	30.510	84.94	33.246	80.76	33.275	0.905

Table S6. Parameters of the S5 sample („end of surgery”) in the three subgroups.

aPTT: activated partial thromboplastin time, BE: base excess, BGA: blood gas analysis, Ca^{2+}: ionized calcium, CCT: conventional coagulation test, CFT: clot formation time, CT: clotting time, FV: factor V, FVII: factor VII, FX: factor X, FXII: factor XIII, INR: international normalized ratio, LT: lysis time, MCF: maximum clot firmness, ML: maximum lysis, PLT: platelet, PT: prothrombin time, SD: standard deviation.

Hyperfibrinolytic group		S3		R3		p
		mean	SD	mean	SD	
BGA	pH	7.40	0.069	7.19	0.189	0.209
	Lactate	2.64	1.007	3	1.445	0.368
	BE	-1.9	3.758	-13.65	7.548	0.044
	Bicarbonate	23.74	2.767	14.88	5.219	0.139
	Ca^{2+}	0.93	0.086	0.46	0.175	0.100
CCT	PLT	159.75	165.673	105.17	131.143	0.120
	PT (s)	27.99	17.025	73	9.147	0.028
	INR	2.5	1.896	6.97	1.083	0,028
	aPTT	70.39	52.909	189.71	5.880	0.028
	Fibrinogen	2.05	1.597	0.2	0.224	0.027
	D-dimer	3.41	1.884	6.1	1.151	0.018
	FV	29.11	21.296	2.7	2.024	0.028
	FVII	46.93	22.758	43.8	26.724	0.735
	FX	42.4	14.779	27.84	18.145	0.091
	FXIII	111	56.254	19.97	8.524	0.018

Table S7. Comparison of systemic (S3) and regional (R3) blood gas and conventional laboratory parameters of anhepatic phase in the hyperfibrinolytic group**.**

aPTT: activated partial thromboplastin time, BE: base excess, BGA: blood gas analysis, Ca^{2+}: ionized calcium, CCT: conventional coagulation test, FV: factor V, FVII: factor VII, FX: factor X, FXII: factor XIII, INR: international normalized ratio, PLT: platelet, PT: prothrombin time, SD: standard deviation.
